# Copaiba Oil Resin Exerts an Additive Effect to Babassu Oil on Behavioral Changes in Human Endometriotic Cell Cultures

**DOI:** 10.3390/ph15111414

**Published:** 2022-11-15

**Authors:** Julianna Henriques da Silva, Leticia Coli Louvisse de Abreu, Renato Ferrari, Celia Yelimar Palmero Quintana, Eliane Gouvêa de Oliveira Barros, Natália de Moraes Cordeiro, Bruno Pontes, Valeria Pereira de Sousa, Lucio Mendes Cabral, Patricia Dias Fernandes, Luiz Eurico Nasciutti

**Affiliations:** 1 Universidade Federal do Rio de Janeiro, Instituto de Ciências Biomédicas, Programa de Pesquisa em Biologia Celular e do Desenvolvimento, Rio de Janeiro 21.941-902, Brazil; 2 Universidade Federal do Rio de Janeiro, Faculdade de Farmácia, Laboratório de Tecnologia Farmacêutica, Rio de Janeiro 21.941-902, Brazil; 3 Universidade Federal do Rio de Janeiro, Hospital Universitário Moncorvo Filho, Instituto de Ginecologia, Rio de Janeiro 20.211-340, Brazil; 4 Universidade Federal do Rio de Janeiro, Instituto de Ciências Biomédicas, Programa de Pesquisa em Descoberta de Fármacos, Laboratório de Farmacologia dor e da Inflamação, Rio de Janeiro 21.941-90, Brazil; 5 Universidade Federal do Rio de Janeiro, Instituto de Ciências Biomédicas, Programa de Pesquisa em Biologia Celular e do Desenvolvimento, Rio de Janeiro 21.941-902, Brazil

**Keywords:** endometriosis, *Orbignya speciosa*, *Copaifera lansgdorffii* nanoemulsion, self-nanoemulsifying drug delivery system

## Abstract

Background: Current drugs for the treatment of endometriosis are not able to completely cure the condition, and significant side effects hinder the continuation of treatment. Therefore, it is necessary to search for new drug candidates. In the present paper, the use of plant extracts is highlighted. Babassu oil and Copaiba oil resin have several therapeutic properties. We investigated the in vitro effects of two nanoemulsions containing oil extracted from Babassu (*Orbignya speciosa*) nuts (called SNEDDS-18) and/or oil resin extracted from Copaiba trunk (*Copaifera langsdorffii*) (called SNEDDS-18/COPA) on cultured human eutopic endometrium stromal cells from endometrial biopsies of patients without (CESC) and with (EuESC) endometriosis as well as human stromal cells from biopsies of endometriotic lesions (EctESC). Methods: CESC, EuESC, and EctESC were taken and treated with SNEDDS-18 and SNEDDS-18/COPA to evaluate their effects on cytotoxicity, cell morphology, proliferation, and signaling pathways. Results: After 48 h of incubation with SNEDDS-18 and SNEDDS-18/COPA, cell viability and proliferation were inhibited, especially in EctESC. The lowest concentration of both nanoemulsions reduced cell viability and proliferation and broke down the cytoskeleton in EctESCs. After 24 h of treatment a decrease in IL-1, TNF-α, and MCP-1 was observed, as well as an increase in IL-10 production. Conclusions: Both nanoemulsions can affect endometriotic stromal cell behaviors, thus revealing two potential candidates for new phytotherapeutic agents for the management of endometriosis.

## 1. Introduction

Endometriosis is one of the most frequent benign gynecological disorders. According to the World Endometriosis Research Foundation, the disease affects 176 million women of reproductive age and 25–50% of all infertile women worldwide [1]. The disease is an estrogen-dependent chronic inflammatory phenomenon characterized by the presence of endometrium-like tissue outside of the uterine cavity. Women with endometriosis generally suffer with dysmenorrhea, chronic pelvic pain, dyspareunia, and infertility, resulting in a compromised quality of life [2,3]. The ideal treatment remains unknown. Moreover, the effectiveness of several drug options is still a source of controversy since none of them can eradicate the foci of the disease [4].

Medicinal plants have become popular for treating the symptoms of various gynecological diseases [5], including endometriosis [6,7]. Copaifera species are widely used in folk medicine. The oil resin obtained from the tree has been used for centuries by indigenous and forest Amazonian people as an anti-inflammatory drug, to cicatrize epidermal wounds, as a urinary antiseptic, and to treat ulcers, bronchitis, and cancer [8]. It has also demonstrated several therapeutic properties, such as anti-inflammatory effects and antitumoral and anti-cell death-inducing activities [9,10,11,12,13]. In relation to endometriosis, a study evaluated the effect of *C. langsdorffii* oil resins and demonstrated a significant reduction in the disease [14].

In Brazilian ethnobotany, Babassu is the popular name of *Orbignya speciosa* Mart. Barbosa Rodrigues (Arecaceae, Palmae). Native to the north, northeast, and central regions of Brazil, ethnopharmacological studies have demonstrated their use for chronic wounds, ulcerations, dysmenorrhea, menstrual pain, constipation, obesity, rheumatism, leukemia, and inflammatory and venous diseases [15,16]. Due to its antiproliferative and apoptotic effects, some studies have suggested Babassu oil as a new and potentially efficient therapy for benign hyperproliferative and inflammatory diseases. Furthermore, it has already been demonstrated that Babassu nanosystems show activity on cell cultures derived from benign prostatic hyperplasia [17]. In nanostructured systems, Babassu also reduced the viability of a macrophage-like cell line and was not toxic to a human colon epithelial cancer cell line, indicating its potential use for the treatment of highly inflammatory processes and for oral administration [18]. Our group demonstrated that a nanocomposite containing Copaiba oil resin induced morphological and behavioral changes in human endometriotic stromal cell cultures, with a decrease in cell viability and proliferation as well as the induction of cell death [19].

It has been widely proposed to apply nanotechnology to plant extracts because nanostructured systems can potentialize the action of these extracts, promoting the controlled release of active constituents, reducing the required dose and side effects, and improving its pharmacological activity [20,21]. Self-nanoemulsifying drug delivery systems (SNEDDS) are used to improve the solubility of hydrophobic drugs and the permeability and oral bioavailability of lipophilic drugs. SNEDDS are isotropic mixtures of oil, surfactant, drug, and cosurfactant [22,23,24].

Given the known pharmacological properties of *O. speciosa* and *C. langsdorffii*, as well as the effectiveness of nanostructured vegetable oils, the aim of this study was to analyze in vitro the effects exerted by two nanoemulsions containing oil extracted from *O. speciosa* nuts (called SNEDDS-18) or containing oil extracted from *O. speciosa* nuts and oil resin extracted from *C. langsdorffii* trunk (called SNEDDS-18/COPA) against the ectopic focus of human endometriotic lesions.

## 2. Results

### 2.1. SNEDDS-18 and SNEDDS-18/COPA Nanoemulsions Preparation

The SNEDDS-18 prepared by our research group resulted in a formulation containing oils, cosurfactants, and surfactants, as described in the materials and methods. The SNEDDS formulations were visually confirmed to be clear, homogeneous solutions that were slightly yellow. When diluted with distilled water to form nanoemulsions, the droplet size (DS) distribution of the resulting emulsions was 43.25 nm, ZP (mv) −18.21 ± 23, and the polydispersity index (PdI) was 0.248. The estimated emulsification time for the SNEDDS-18 formulation was 1 min and 32 s, which classifies this formulation as a class B microemulsion system. It was observed that SNEDDS-18 should accept a volume of water of greater than 200% of initial volume, which indicates the strong possibility of nanoemulsion formation in vivo. The Babassu oil content in the formulation was confirmed using a validated GC-MS analytical methodology, with lauric acid being measured as a chemical marker of the plant drug [23].

### 2.2. SNEDDS-18 and SNEDDS-18/COPA Does Not Show Toxicity In Vivo

Daily oral treatment of mice with 100 mg/kg of both nanoformulations did not induce either behavioral alteration nor signs of intoxication. Post-mortem examination did not demonstrate gastric lesions, bleeding, or hematological alterations (data not shown).

### 2.3. SNEDDS-18 and SNEDDS-18/COPA Reduce Cell Viability in EctESC Cultures

**Figure 1:**
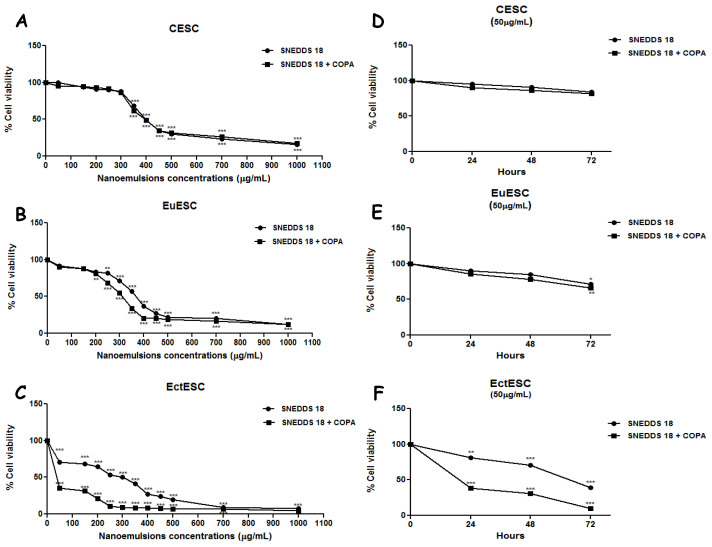
Endometrial stromal cell viability after incubation with nanoemulsions. In (**A**–**C**), CESC, EuESC, and EctESC were treated with different concentrations of SNEDDS-18 or SNEDDS-18/COPA for 48 h in complete medium. In (**D**–**F**) CESC, EuESC, and EctESC were treated with 50 µg/mL of SNEDDS-18 or SNEDDS-18/COPA for 24, 48, or 72 h. Cell viability was analyzed by the MTT method. Data are expressed as mean ± standard deviation of cell viability (in %). * *p* < 0.05; ** *p* < 0.01; *** *p* < 0.001 when comparing nanoformulation-treated groups with nontreated group. CESC: Endometrial stromal cells obtained from topic endometrium of patients without endometriosis; EuESC: Endometrial stromal cells obtained from topic endometrium of endometriosis patients; EctESC: Endometrial stromal cells obtained from ectopic endometrium of endometriosis patients (endometriotic lesions); SNEDDS-18: Nanoemulsion containing Babassu oil; SNEDDS-18/COPA: nanoemulsion containing Babassu oil and Copaiba oil resin.

Incubation of CESC, EuESC, or EctESC human endometrium stromal cells over 48 h with increasing concentrations of SNEDDS-18 or SNEEDDS/COPA resulted in a dose-dependent effect (Figure 1A–C). It is interesting to note that EctESC was the most sensitive to cell death induced by each one of the nanoformulations (Figure 1C). We next evaluated the effects of SNEDDS-18 and SNEDDS-18/COPA (at 50 µg/mL) against the three different cells incubated for 24, 48, or 72 h. It is evident that even after 72 h of incubation with CESC or EuESC cells with nanoformulations (at 50 µg/mL), no reduction in cell viability was observed. On the other hand, EctESC cells showed drastically reduced viability after 24 h of incubation (Figure 1F).

### 2.4. SNEDDS-18 and SNEDDS-18/COPA Reduce Cell Proliferation in EctESC Cultures

**Figure 2:**
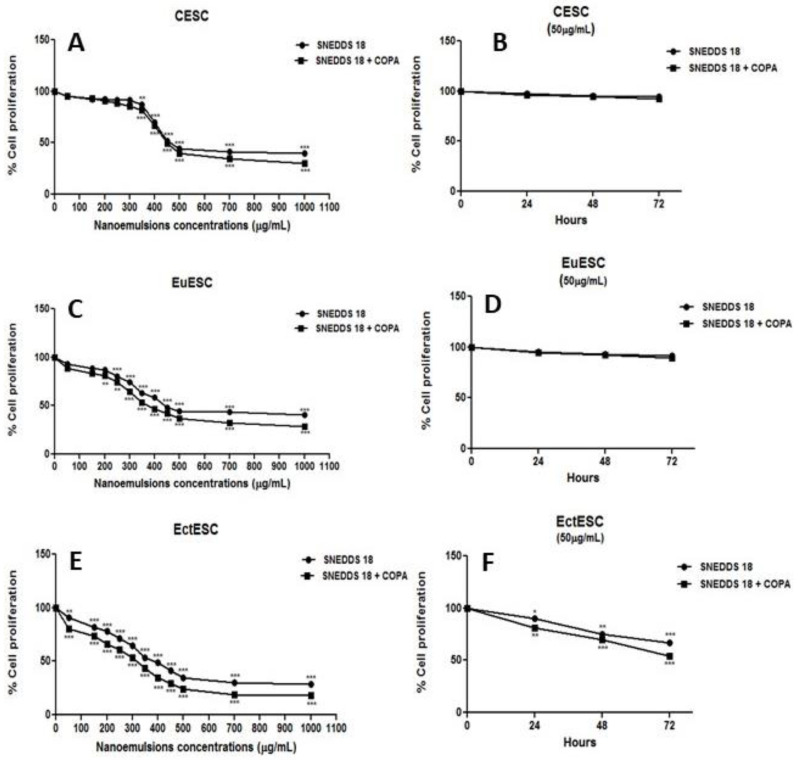
Endometrial stromal cell viability after incubation with nanoemulsions. In (**A**), (**B**), and (**C**), CESC, EuESC, and EctESC were treated with different concentrations of SNEDDS-18 or SNEDDS-18/COPA for 48 h in complete medium. In (**D**), (**E**), and (**F**), CESC, EuESC, and EctESC were treated with 50 µg/mL of SNEDDS-18 or SNEDDS-18/COPA for 24, 48, or 72 h. Cell proliferation was analyzed by crystal violet method. Data are expressed as mean ± standard deviation of cell viability (in %). * *p* < 0.05; ** *p* < 0.01; *** *p* < 0.001 when comparing nanoformulation-treated groups with nontreated group. CESC: Endometrial stromal cells obtained from topic endometrium of patients without endometriosis; EuESC: Endometrial stromal cells obtained from topic endometrium of endometriosis patients; EctESC: Endometrial stromal cells obtained from ectopic endometrium of endometriosis patients (endometriotic lesions); SNEDDS-18: Nanoemulsion containing Babassu oil; SNEDDS-18/COPA: nanoemulsion containing Babassu oil and Copaiba oil resin.

Considering the proliferative nature of endometriosis, we investigated whether both formulations could interfere with the proliferative status of the endometrial stromal cell cultures. Figure 2A, B, C show that different concentrations of both nanoemulsions significantly reduce, in a dose-dependent manner, the proliferative activity of CESC, EuESC, and EctESC. It is worth highlighting that EctESCs proliferation was significantly decreased over the treatment period (Figure 2F).

**Figure 3:**
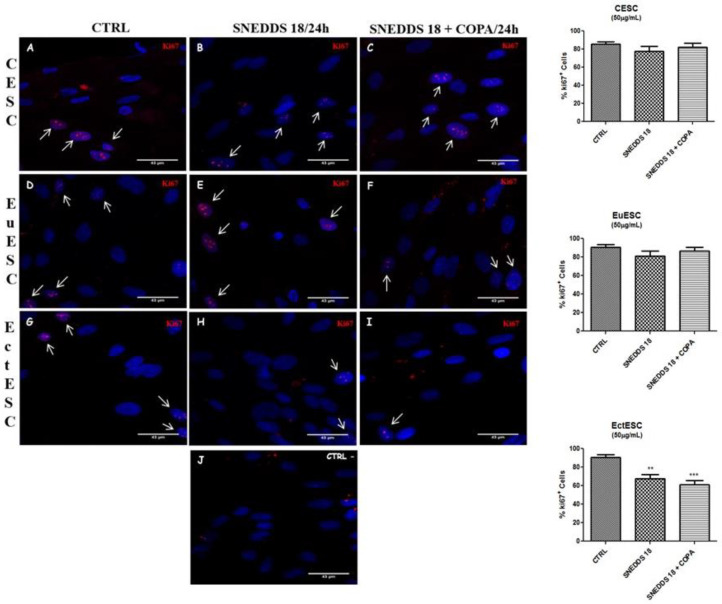
Immunostaining of Ki-67-positive cells (left photos). CESC (**A**–**C**), EuESC (**D**–**F**), and EctESC (**G**–**I**) were incubated with Ki-67 and treated or left untreated with SNEDDS-18 or SNEDDS-18/COPA (50 µg/mL) for 24 h. In (**J**), the negative reaction is shown. In red is Ki-67 labelling, and in blue is DAPI (nuclear marker) labelling. Representative images of three independent experiments performed in triplicate. Scale bar = 43 μm. Percentage of Ki-67-positive cells (right graphs). Results are expressed as mean ± SD. ** *p* < 0.01; *** *p* < 0.001 when comparing SNEDDS-18 or SNEDDS-18/COPA with vehicle group. CESC: Endometrial stromal cells obtained from topic endometrium of patients without endometriosis; EuESC: Endometrial stromal cells obtained from topic endometrium of endometriosis patients; EctESC: Endometrial stromal cells obtained from ectopic endometrium of endometriosis patients (endometriotic lesions); SNEDDS-18: Nanoemulsion containing Babassu oil; SNEDDS-18/COPA: nanoemulsion containing Babassu oil and Copaiba oil resin.

To confirm the reduction in cell proliferative activity induced by SNEDDS-18 and SNEDDS-18/COPA, we performed immunostaining for the cell proliferation antigen Ki-67. In control groups (CESCs, EuESCs, and EctESCs without treatment), it is possible to observe Ki-67-positive immunostaining (strong or faint, in red) (Figure 3A–I, white arrows). After 24 h of SNEDDS-18 and SNEDDS-18/COPA treatments (50 μg/mL), it can be observed that CESC and EuESC cultures preserved a strong immunostaining for Ki-67 (Figure 3A–C and 3D–F, respectively). In contrast, EctESC cultures exhibited a reduction in Ki-67-positive staining (Figure 3G–I). When quantifying the percentage of positive Ki-67 cells, EctESC cells treated with SNEDDS-18 or SNEDDS-18/COPA exhibited a reduction of 30% and 38% in cell proliferation, respectively (Figure 3, right graphs).

### 2.5. SNEDDS-18 and SNEDDS-18/COPA Alters EctESC Motility

**Figure 4:**
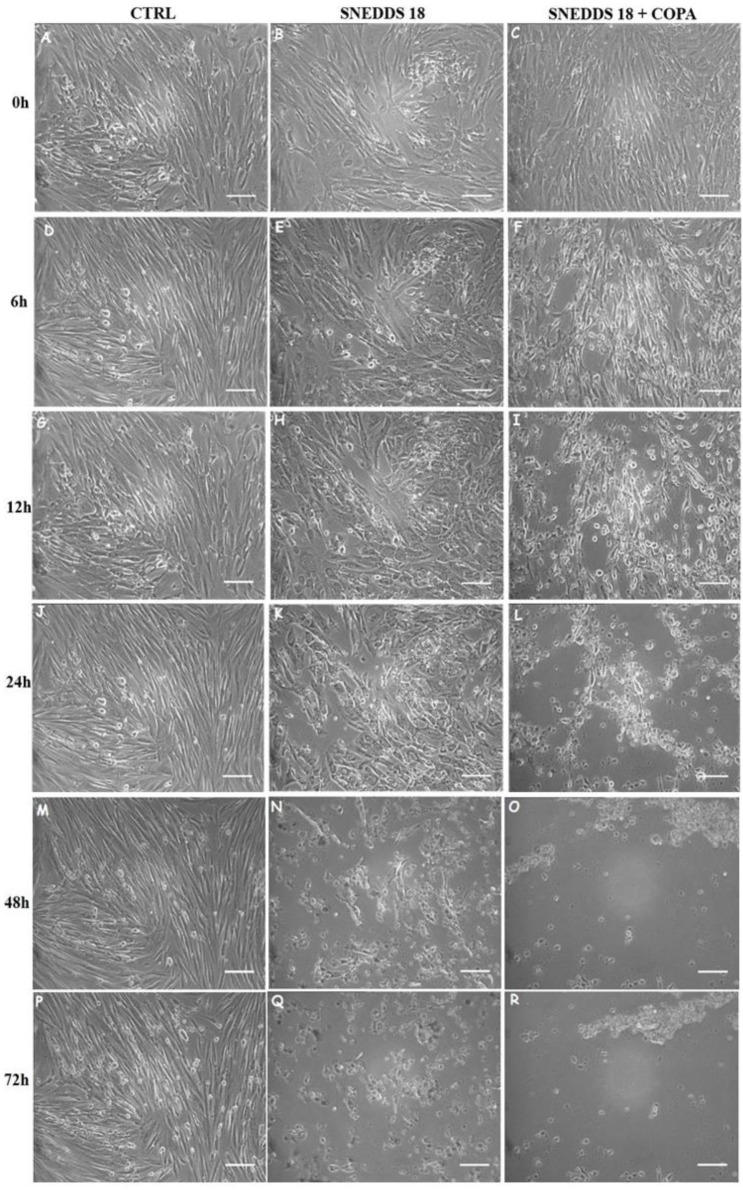
Alterations in EctESC cultures after treatment with SNEDDS-18 and SNEDDS-18/COPA. A-R are representative images of untreated and treated EctESC after incubation with nanoemulsions of SNEDDS-18 and SNEDDS-18/COPA (50 μg/mL) obtained by phase contrast microscopy at 0 (**A**–**C**), 6 (**D**–**F**), 12 (**G**–**I**), 24 (**J**–**L**), 48 (**M**–**O**), and 72 (**P**–**R**) h. Scale bar = 50 μm. CESC: Endometrial stromal cells obtained from topic endometrium of patients without endometriosis; EuESC: Endometrial stromal cells obtained from topic endometrium of endometriosis patients; EctESC: Endometrial stromal cells obtained from ectopic endometrium of endometriosis patients (endometriotic lesions). SNEDDS-18: Nanoemulsion containing Babassu oil. SNEDDS-18/COPA: Nanoemulsion containing Babassu oil and Copaiba oil resin.

Next, we asked whether SNEDDS-18 and SNEDDS-18/COPA could influence cell motility. CESC, EuESC, and EctESC motility were evaluated through videomicroscopy after 72 h of exposure to 50 μg/mL of SNEDDS-18 or SNEDDS-18/COPA. It was observed that the cells exhibited an elongated and spread morphology with intense proliferation, metabolic activity, and accelerated cell movement. No significant differences in motility were found between the three untreated cell cultures (data not shown). On the other hand, when CESCs, EuESCs, and EctESCs were treated with SNEDDS/COPA, only the last ones presented morphological alterations and reduced motility. Notably, over the 72 h, we observed several events indicative of cell damage and death in EctESC cultures treated with both SNEDDS-18 and SNEDDS-18/COPA, such as plasma membrane and cytoplasm retraction, loss of cell–cell contact, loss of cell–substrate adhesion, and presence of intracytoplasmic vacuoles, leading to the deconfiguration of the EctESC monolayer. In fact, within the first 6 h of treatment with SNEDDS-18/COPA, we observed intense cellular breakdown, culminating in the disruption of the EctESC monolayer (Figure 4).

### 2.6. Nanoemulsions Disturbs the Cytoskeleton of the EctESCs Cultures

**Figure 5:**
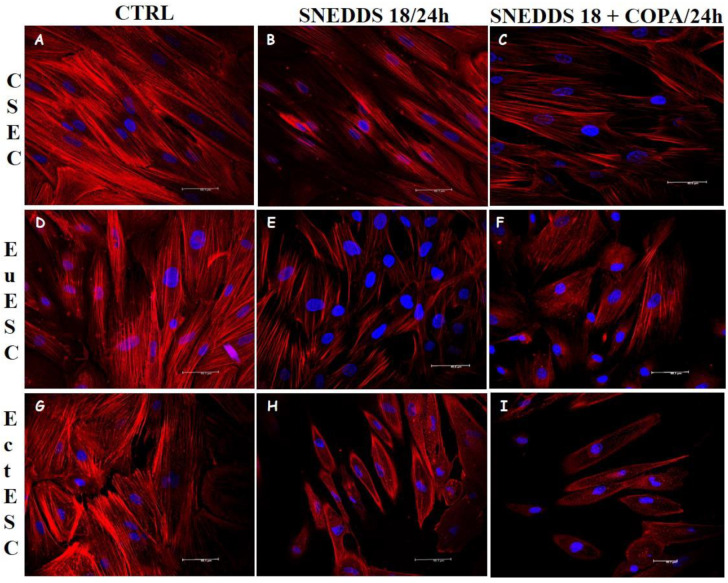
Alterations in actin filaments of ESC cultures after treatment with nanoemulsions. CESC (**A**–**C**), EuESC (**D**–**F**), and EctESC (**G**–**I**) were labeled with Cy3 fluorochrome-coupled fungal phalloidin toxin (rhodamine, red) or DAPI (nuclear marker, blue) 24 h after treatment with nanoemulsions. Representative images of three independent experiments performed in triplicate. Scale bar = 50 μm. CESC: Endometrial stromal cells obtained from topic endometrium of patients without endometriosis; EuESC: Endometrial stromal cells obtained from topic endometrium of endometriosis patients; EctESC: Endometrial stromal cells obtained from ectopic endometrium of endometriosis patients (endometriotic lesions).

In order to better understand the influence of the nanoemulsions on the structure of the stromal cells, we analyzed the organization of the three cytoskeleton components (microfilaments, microtubules, and intermediate filaments) after 24 h of treatment with the nanoemulsions. In control conditions, actin filaments are polymerized and organized in parallel to each other, giving the cells their scattered morphology, providing the necessary support to the plasma membrane, and allowing cell–cell and cell–substrate adhesion. (Figure 5).

**Figure 6:**
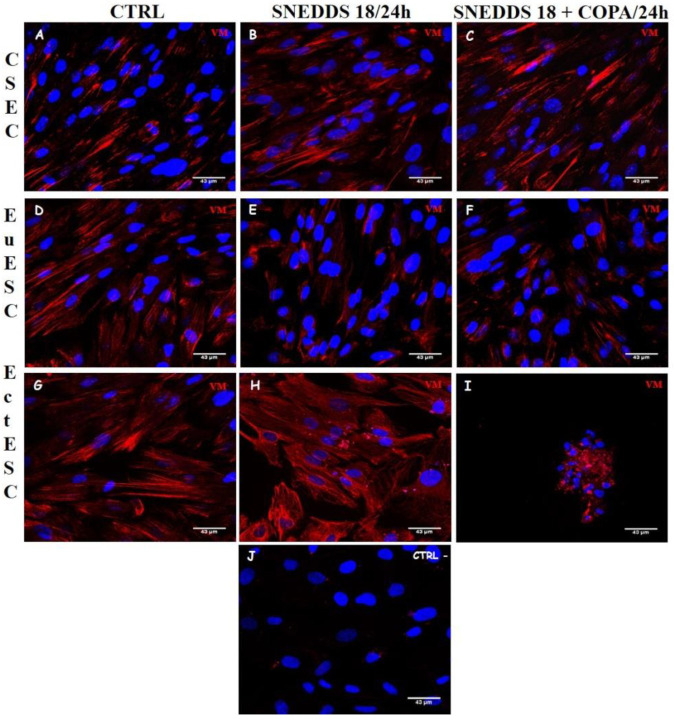
Morphological analysis of vimentin intermediate filaments of the ESC cultures treated with SNEDDS-18 and SNEDDS-18/COPA. CESC (**A**–**C**), EuESC (**D**–**F**), and EctESC (**G**–**I**) were labeled with anti-vimentin antibody (red) and DAPI (**J**, nuclear marking—blue) after 24 h of treatment with nanoemulsions (50 µg/mL). Representative images of three independent experiments performed in triplicate. Scale bar = 43 μm. CESC: Endometrial stromal cells obtained from topic endometrium of patients without endometriosis; EuESC: Endometrial stromal cells obtained from topic endometrium of endometriosis patients; EctESC: Endometrial stromal cells obtained from ectopic endometrium of endometriosis patients (endometriotic lesions).

Vimentin is a protein of the intermediate filament family, and together with actin microfilaments and microtubules it is part of the cytoskeleton. This protein is present in cells of mesodermal origin, functioning as a marker of stromal cells, and for that reason its presence and distribution were also analyzed in cell cultures treated with both nanoformulations. In the untreated CESC, EuESC, and EctESC cultures, we observed vimentin filaments distributed through the cytoplasm, organized in stable protein networks, forming structures similar to strings or wire ropes, conferring mechanical strength and stability to these cells. Likewise, we observed that treated CESC and EuESC cultures preserved their vimentin cytoskeleton, while in treated EctESC cultures, all this organization was lost. It is important to note that when EctESCs were treated with SNEDDS-18/COPA, this effect appears to be exacerbated, and the entire intermediate vimentin cytoskeleton is undone, indicating a probable additive effect of Copaiba oil resin to Babassu oil (Figure 6).

**Figure 7:**
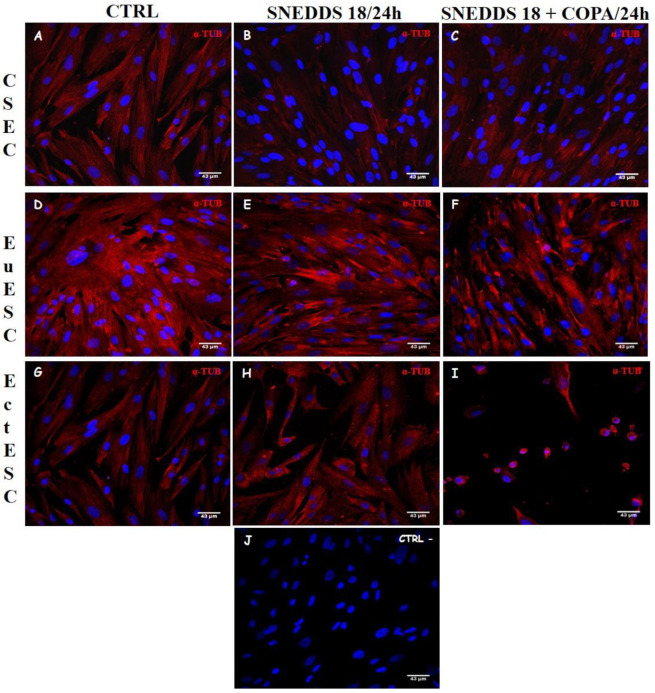
Analysis of tubulin structure of the ESC cultures after treatment with nanoemulsions. CESC (**A**–**C**), EuESC (**D**–**E**), and EctESC (**G**–**I**) were labeled with anti-α-tubulin antibody (red) or DAPI (**J**, nuclear marker, blue) after 24 h of treatment with nanoemulsions (50 µg/mL). Scale bar 43 = μm. CESC: Endometrial stromal cells obtained from topic endometrium of patients without endometriosis; EuESC: Endometrial stromal cells obtained from topic endometrium of endometriosis patients; EctESC: Endometrial stromal cells obtained from ectopic endometrium of endometriosis patients (endometriotic lesions).

Figure 7 shows that untreated CESC, EuESC, and EctESC cultures presented an organized α-tubulin cytoskeleton, creating the polarized, elongated, and scattered characteristic morphology of these stromal cell cultures. After 24 h of treatment with both nanoformulations, the CESC and EuESC cultures preserved the structure of the microtubule cytoskeleton. In contrast, a clear disruption of protofilaments can be observed in EctESC cultures, culminating in cytoplasmic retraction and, consequently, changes in their morphology.

### 2.7. Nanoemulsions Induce Changes in Cell Adhesion Process in EctESC Cultures

**Figure 8:**
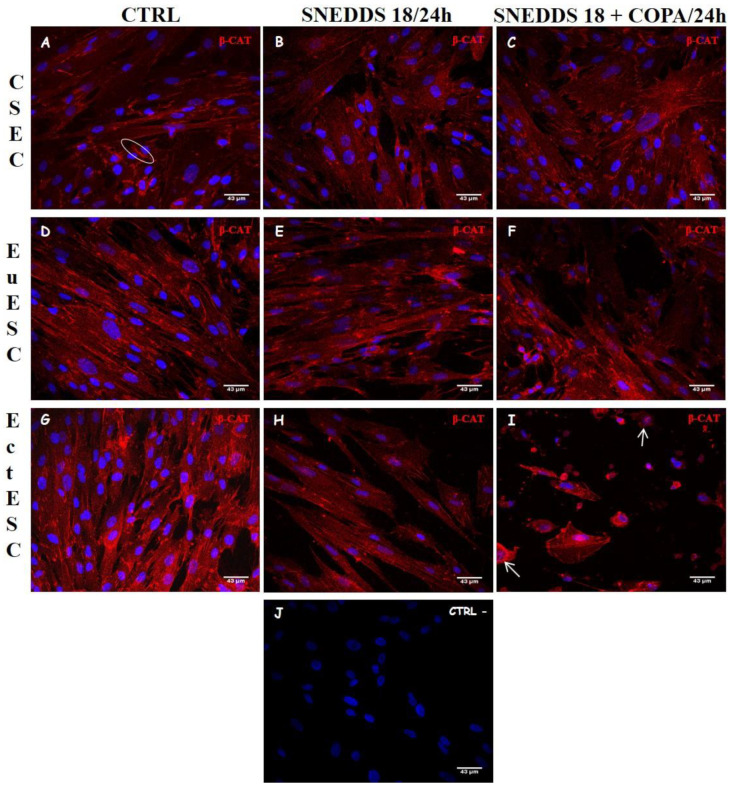
Distribution of β-catenin on ESC cultures treated nanoemulsions by immunocytochemistry using anti-β-catenin antibody. CESC (**A**–**C**), EuESC (**D**–**E**), and EctESC (**G**–**I**) were labeled with anti-β-catenin antibody and DAPI (**J**, nuclear marking, blue) after 24 h of treatment with nanoformulations (50 µg/mL). Representative images of three independent experiments performed in triplicate. Scale bar = 43 μm. CESC: Endometrial stromal cells obtained from topic endometrium of patients without endometriosis; EuESC: Endometrial stromal cells obtained from topic endometrium of endometriosis patients; EctESC: Endometrial stromal cells obtained from ectopic endometrium of endometriosis patients (endometriotic lesions).

Due to the structural changes observed, we decided to evaluate the extent to which both nanoemulsions could interfere with the cell adhesion process. For this, we evaluated the distribution of β-catenin, an integral structural component of cadherin-based adherens junctions. In the untreated CESC, EuESC, and EctESC cultures, the location and distribution of β-catenin are preserved, and the protein remains located in the cell cytoplasm (as fine granules), mainly in the plasma membrane at the points of cell–cell adhesion. After 24 h of treatment with nanoemulsions, we noticed that only the cultures of EctESCs presented changes in the distribution and location of β-catenin, which concentrated on the cell cytoplasm. A remarkable nuclear localization of β-catenin was also observed EctESCs treated with SNEDDS-18/COPA. Thus, both SNEDDS-18 and SNEDDS-18/COPA modified the distribution of β-catenin exclusively in EctESC cultures (Figure 8, white arrows).

### 2.8. SNEDDS-18 and SNEDDS-18/COPA Affect the Secretion of Cytokines by EctESC Cultures

**Figure 9:**
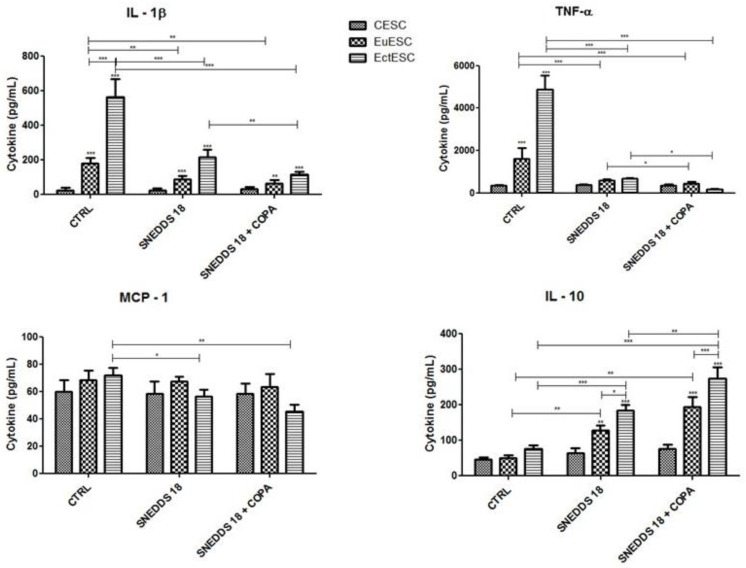
Production of cytokines by CESC, EuESC, and EctESC cells after treatment with nanoemulsions. After 24 h of incubation with nanoemulsions (50 µg/mL), supernatants were collected to measurement of indicated cytokines. Results are expressed as mean ± SD (*n* = 9). * *p* < 0.05; ** *p* < 0.01; *** *p* < 0.001 when comparing CESC-, EuESC-, and EctESC-nanoemulsion-treated groups with vehicle-treated group. CESC: Endometrial stromal cells obtained from topic endometrium of patients without endometriosis; EuESC: Endometrial stromal cells obtained from topic endometrium of endometriosis patients; EctESC: Endometrial stromal cells obtained from ectopic endometrium of endometriosis patients (endometriotic lesions).

Next, we evaluated the effects of nanoemulsions in regulating cytokine production by cell cultures. CESC, EuESC, and EctESC were treated with SNEDDS-18 and SNEDDS-18/COPA, and production of IL-1β, TNF-α, IL-10, and MCP-1 was analyzed. EctESC cells did secrete more cytokines than cell cultures obtained from topical endometrium’s (CESC and EuESC). After 24 h of treatment with one of the nanoemulsions, there was a significant decrease in the secretion of IL-1β and TNF-α in the EuESC and EctESC cultures. As expected, SNEDDS-18/COPA reduced IL-1β secretion more intensely in EctESC cultures. (Figure 9). No significant differences were observed between CESC, EuESC, and EctESC untreated cultures. In contrast, both treatments decreased MCP-1 secretion in EctESC cultures. On the other hand, nanoemulsions increased IL-10 production by EuESC and EctESC cultures. Moreover, we observed that SNEDDS-18 and especially SNEDDS-18/COPA increased the secretion of IL-10 by EctESC cultures significantly when compared to EuESCs (Figure 9).

## 3. Discussion

Endometriosis is a benign, chronic, and inflammatory gynecological condition commonly seen in women of reproductive age [25]. Although much progress has been made in its treatment, there is a lack of direct drugs with few side effects. Given this scenario, the discovery of new therapeutic alternatives is necessary.

Plants and herbal medicines stand out as important sources of new molecules and bioactive compounds with pharmacological activity. Both Copaiba oil resin and Babassu oil have extensive popular use in traditional medicine. Copaiba-exuded oleoresin is used as an anti-inflammatory, to heal wounds, as a urinary antiseptic, and to treat ulcers, bronchitis, and cancer [8]. Pharmacological studies have confirmed its anti-inflammatory [8,10,13,18,26], antitumoral [12,27,28], and analgesic [12] effects.

In this work, we analyzed the effects of two nanoformulations containing Babassu oil (*Orbignya speciosa*) and Copaiba oil resin (*Copaifera langsdorffii*) on CESC, EuESC, and EctESC cell cultures obtained from normal or ectopic focus of human endometriotic lesions.

The role of nanometric systems as therapeutic carriers has been extensively investigated. In fact, the application of nanomedicine is increasing, with the promise of improving the efficiency of substance retention in lesions and the optimization of pharmacokinetic properties [29]. In addition, nanostructured systems favor the in vitro immunomodulatory response of the peritoneal macrophages in women with endometriosis [30], in addition to compromising the behavior of cell cultures taken from human endometriotic lesions, reducing cell viability, cell proliferation, and altering the cell morphology [19]. Moreover, the in vivo inhibition of endometriosis progression via regulation of murine peritoneal macrophages has also been shown [31]. Many studies address phytotherapy as a therapeutic alternative for endometriosis treatment. In this vein, some studies corroborate our findings, evidencing the antiendometriotic effects of various extracts and plant actives [32,33,34,35,36,37,38,39,40]. Likewise, our results are in accordance with a reduction in cell viability as found by Cao et al. [41], as well as a decrease in the expression of several molecules involved in the pathophysiology of endometriosis (VCAM-1, ICAM- 1, TNF-α, IL-1, IL-6, IL-8, and MCP-1) in cell cultures obtained from endometriotic lesions after treatment with curcumin [36].

There are a few reported cases in the literature that analyze the treatment of endometriosis with the oil extract from Copaiba [14,19], but there are practically no studies evaluating the effects of Babassu oil or extract on this gynecological disorder. Our research group had already demonstrated that a nanocomposite containing oil resin from Copaiba (*C. langsdorffii*) modified cell cultures obtained from endometriotic lesions of the ovary capsule; in this case, the viability of these cell was reduced to 46% [19]. We observed that the nanoemulsion composed of the oil extracted from both species demonstrated a more intense effect than the nanoemulsion consisting only of Babassu oil, suggesting a possible additive effect of the two combined. These results are corroborated by other studies in which vegetable preparations consisting of several types of medicinal plants with antiendometriotic properties [42,43,44] demonstrated a greater effect when the assets were combined in the same preparation [43,45]. Our analysis allowed us to verify that the nanoformulations reduced the viability and proliferation of EctESCs without compromising the behavior of CESCs or EuESCs. It is important to note that the cell cultures from topical endometrium (CESC and EuESC) showed no change in monolayer configuration after treatment. Corroborating these results, other studies have shown a reduction in contractility [46], invasive capacity [33,47,48], and cell migration [36,38] of human endometriotic cell cultures after therapeutic treatment. Our findings further indicate that different behaviors are exhibited by the stromal cell cultures obtained from the topical and ectopic endometrium. Additionally, changes in the cell morphology of EctESCs treated with SNEDDS-18/COPA were clearly observed in which the three strands of the cell cytoskeleton (actin, α-tubulin, and vimentin) were disorganized and unstructured. It is possible that the disorganization of these cytoskeleton filaments is associated with the reduction in the viability of EctESC cultures induced by both nanoemulsions. Corroborating our analyses, studies have shown a significant reduction in cell viability and changes in the morphology of the endometriotic cell cultures treated with different extracts, oils, and plant formulations [19,33,36,37,46].

Interestingly, treatment with nanoemulsions reduced the proliferative activity of EctESCs. These data are consistent with other studies on phytotherapy and endometriosis [35,37,49,50,51,52]. In this context, Wieser et al. [52] and Zhou and Qu [44] reported proliferation inhibition and apoptosis induction of the stromal cell cultures derived from endometriotic lesions by a Chinese preparation of nine medicinal plants with antiendometriotic properties. Furthermore, confirming our analyses, Lian et al. [42] evaluated the effectiveness of the Quyu Jiedu Recipe (QJR), a Chinese herbal preparation, in the treatment of endometriosis, and revealed that this formula was able to significantly reduce VEGF expression and the cell proliferation antigen Ki-67.

The multifunctional protein β-catenin is one of the most important binding partners in the cell–cell adhesion process, in addition to being the central signal-transducing molecule of the WNT pathway. When located in the plasma membrane, β-catenin participates in cell–cell adhesion. Our data indicated that both nanoemulsions modified the distribution and location of β-catenin exclusively in EctESC cultures, inducing the translocation of β-catenin from the plasma membrane to the cytoplasm and resulting in intense loss of cell–cell adhesion, morphologic changes, and decreasing cell viability and cell proliferation. Corroborating our results, several studies revealed a decrease in the expression of adhesion proteins, induced by therapeutic strategies, which compromised the development and progression of endometriosis in vivo and in vitro [36,53,54,55,56].

Endometriosis is a chronic inflammatory disease with the production of several cytokines [57,58,59]. An increase in the amount of these cytokines has also been observed in the peritoneal fluid of patients with endometriosis [57,59,60,61,62,63,64]. Our data indicate that EctESCs released a greater amount of IL-1β and TNF-α than EuESC and CESC, and both nanoemulsions significantly reduced IL-1β and TNF-α secretion in EuESCs and EctESCs. This is in line with an additive effect of Copaiba oil resin to Babassu oil in the pathogenesis of endometriosis. We also showed that the combination of both oils (Babassu and Copaiba) decreased MCP-1 secretion and increased IL-10 production by CESCs, EuESCs, and EctESCs. High concentrations of MCP-1 have been detected in the peritoneal fluid of women with endometriosis and in in vitro and in vivo models [65,66,67]. IL-10 is an immunomodulatory cytokine that can inhibit the synthesis of cytokines such as Interferon-γ (IFN-γ), IL-2, IL-3, and TNF-α [68]. Interestingly, studies have revealed elevated levels of IL-10 in the peritoneal fluid of women with advanced endometriosis [69,70,71]. Thus, local deregulation of cytokines allows endometrial fragments to adhere and implant in the peritoneal cavity.

Taken together, our data are extremely relevant for the therapeutic treatment of endometriosis since the existence of a compound that can reduce the inflammation component in both the uterine (topical endometrium) and extrauterine (ectopic endometrium) environment would assist in the control of endometriosis growth and progression.

## 4. Materials and Methods

### 4.1. Preparation of SNEDDS-18 and SNEDDS-18/COPA

BBS (*Orbignya speciosa*) and COPA (*Copaifera landsdorffi*) oils were supplied by To-basa Bioindustrial of Babaçu S.A. (Tocantins, Brazil) and Imaflora—Instituto de Manejo e Certificação Florestal e Agrícola (SP, Brazil), respectively. Once developed in Laboratório de Tecnologia Industrial Farmacêutica, Pharmacy Scholl Federal University of Rio de Janeiro, SNEDDS-18 and SNEDDS-18/COPA were prepared according to a protocol described by our research group [23], which included a fractional factorial design approach (Type 24) that generated 25 different formulations. Babassu oil (from *O. speciosa*), Copaiba oil resin (from *C. langsdorffi*), oleic acid (Sigma Aldrich, Burlington, MA, USA), oleylamine (Sigma Aldrich, USA), Labrafac^®^ (Gattefossé, France), Labrasol^®^ (Gattefossé, France), and isopropyl myristate (Vetec, Brazil) were used as components of the oil phase that was combined with the cosurfactants transcutol HP (Sigma Aldrich, USA), ethanol, and propyleneglycol. Surfactants consisted of Tween^®^ 20 (Vetec, Rio de Janeiro, Brazil), Tween^®^ 80 (Vetec, Brazil), Span^®^ 80 (Vetec, Brazil), lecithin (Sigma Aldrich, USA), and Kolliphor RH 40 (Sigma Aldrich, USA). The surfactants were mixed by magnetic stirring before the oil/cosurfactant phase was added. To evaluate the optimal conditions for formulation preparation, pseudoternary phase diagrams were developed through an aqueous titration method [23]. For each diagram, the surfactant and cosurfactant (S/CoS) were mixed at approximately a 5:2 wt ratio. Next, the oil phase was added to the preformed S/CoS phase at different weight ratios (9:1, 8:2, 7:3, 6:4, 5:5, 4:6, 3:7, 2:8, and 1:9). Subsequently, each mixture was titrated with water by the progressive addition of 10 μL volumes and visually observed for phase clarity and viscosity. CHEMIX School software v3.51 (Arne Standnes, Bergen, Norway) was used to construct the diagrams. The obtained SNEDDS, characterized by robustness for dilution, emulsification time, thermodynamic stability, and droplet size and oil content stability studies for 3 months at 40 °C, were diluted directly in Dulbecco’s Modified Eagle’s Medium (DMEM, Invitrogen, Waltham, MA, USA) supplemented with 10% fetal bovine serum (hereafter called complete medium), to provide working solutions (50 or and 1000 µg/mL).

### 4.2. Endometrial Tissue and Endometriotic Lesion Samples

This study was approved (protocol no. 23002513.7.0000.5257, 2014) by the Research Ethics Committee of Hospital Universitário Clementino Fraga Filho of the Federal University of Rio de Janeiro/Brazil. Samples of normal endometrial tissue were obtained from three patients without endometriosis submitting to a total hysterectomy for myoma treatment. Samples of topic endometrium and endometriotic lesions were collected from three patients with endometriosis undergoing videolaparoscopy. All tissue samples were isolated according to Olivares et al. [72].

### 4.3. Acute Toxicity Test

The toxicity assays were performed on male and female Swiss Webster mice (18–25 g). The animals were kept in a temperature-controlled room (22 ± 2 °C) in light/dark cycles for 12 h, with free access to food and water. Animal care and research protocols were in accordance with the principles and guidelines adopted by the Brazilian College of Animal Experimentation. The protocol for animal use was approved (code DFBCICB-015) by the animal experimentation ethics committee of the Health Sciences Center of UFRJ, Rio de Janeiro. Parameters of acute toxicity were determined according to Lorke [72], with some modifications. The daily oral dose (100 mg/kg) of the two nanoemulsions (SNEDDS-18 and SNEDDS-18/COPA) was administered separately to groups of 15 male and female mice for 15 consecutive days. Daily, after 0.5, 1, 4, 6, and 24 h, Lorke’s parameters were observed. After the fifteenth day, the animals were sacrificed by cervical dislocation, stomachs removed, and an incision along the greater curvature was made. Next, the number of ulcers (single or multiple erosions, ulcers, or perforations) and hyperemic areas were counted, and blood samples were collected by the tails and analyzed on a CellPouch automatic counter (pocH-100iV Diff, Sysmex, Kobe, Japan).

### 4.4. Cell Viability Assay

Cellular survival was obtained using the 3-[4,5-dimethylthiazol-2y]-2,5-diphenyltertrazolium bromide (MTT) assay. Cells (5 × 10^3^) obtained from CESCs, EuESCs, and EctESCs were seeded into a 96-well cell culture plate and exposed for 48 h to different concentrations of SNEDDS-18 and SNEDDS-18/COPA. After treatment, a MTT solution (0.5 mg/mL) was added, and after 4 h of incubation at 37 °C, supernatant was discarded and dimethylsulfoxide was added. Formazan crystals formed were quantified at 570 nm in a spectrophotometer (BIO-RAD iMARKE, Hercules, CA, USA).

### 4.5. Evaluation of Cell Morphology and Proliferation

Morphological changes and cellular proliferation were assessed using laser scanning confocal microscopy. CESCs, EuESCs, and EctESCs (1 × 10^4^) were seeded on 12 mm round coverslips and treated with SNEDDS-18 and SNEDDS-18/COPA (50 µg/mL) for 24 h. Subsequently, the cells were washed with sterile PBS and fixed with cold ethanol for 20 min at room temperature. To assess intermediate filament vimentin and microtubules, the cells were incubated with the monoclonal primary antibodies anti-vimentin (1:100 dilution, Sigma-Aldrich) or anti-α-tubulin (1:100 dilution, Sigma-Aldrich) for 2 h at room temperature, followed by an additional 2 h of incubation with Alexa 546 secondary antibody (1:300 dilution, Invitrogen). Actin microfilaments were evaluated by staining with phalloidin conjugated to rhodamine (1:100 dilution, Sigma) for 60 min at room temperature. To detect Ki-67, the cells were incubated with the Ki-67 antibody (1:100 dilution, Dako) for 18 h at room temperature, followed by Alexa 546 secondary antibody (1:300 dilution, Invitrogen). Cell nuclei were labeled with DAPI (5 µg/mL, Santa Cruz Biotechnology, Santa Cruz, CA, USA). Coverslips were mounted onto histological slides with N-propylgalate (Sigma-Aldrich) and images obtained by fluorescence microscopy (Nikon, Tokyo, Japan) attached to a digital camera (Coolpix 990; Nikon). For each case, immunostaining-negative controls were performed by omitting the primary antibody. The images shown are representative of at least three separate experiments.

The possible effects of both nanoemulsions on cell proliferation was also assessed by the crystal violet incorporation assay. The three ESC cultures (5 × 10^3^) were seeded and treated for 24, 48, and 72 h. Then the cells were washed, fixed in ethanol for 10 min, stained with crystal violet 0.05% (Vetec, Brazil) for 10 min, and solubilized with methanol. The supernatant was measured by spectrophotometer (BIO-RAD iMARKE) at 570 nm. All assays were performed in triplicate.

### 4.6. Analysis of the Cellular Motility

To evaluate the in vitro motility of the CESCs, EuESCs, and EctESCs over the 72 h of treatment with 50 μg/mL of SNEDDS-18 and SNEDDS-18/COPA, we used videomicroscopy assay. To do that, each cell culture was plated, and samples were moved to a culture chamber with controlled conditions of temperature and CO_2_ (37 °C and 5%, respectively), adapted to an inverted microscope Nikon Eclipse TE 300 (Nikon). Over the 72 h, phase contrast images of treated and untreated cell cultures were captured every minute using a Hamamatsu C2400 CCD camera (Hamamatsu, Japan), and later, movies were assembled.

### 4.7. Analysis of Cellular Adhesion

To determine whether the nanoemulsions could interfere with cellular adhesion, we performed a cell adhesion test by immunocytochemistry, as described for morphological analysis. Briefly, the CESC, EuESC, and EctESC cultures in equal number (1 × 10^4^ cells/well) were treated for 24 h, fixed, permeabilized, and incubated with anti-β-catenin primary antibody (1:100, Sigma-Aldrich) followed by Alexa 546 secondary antibody (1:300, Invitrogen). Then, the cells were incubated with DAPI, the coverslips mounted onto histological slides and photographed in a fluorescence microscope (Nikon, Tokyo, Japan). The images shown are representative of at least three separate experiments.

### 4.8. Cytokine Measurement

Conditioned medium was obtained 24 h after incubation of the CESCs, EuESCs, and EctESCs with 50 µg/mL of nanoemulsions (SNEDDS-18 and SNEDDS-18/COPA). Specific ELISA kits were used (BD OptEIA™ Set Mouse ELISA) for IL-1β, TNF-α, MCP-1, and IL-10, and their levels were determined according to the manufacturer’s recommendations (BD Biosciences, Haryana, India). Absorbance was measured at 450 nm using a microplate reader (BIO-RAD), and cytokine concentrations were calculated using a standard curve.

### 4.9. Data Analysis

The statistical analyses were performed using the GraphPad Prism 5.0 (GraphPad Software Inc., San Diego, CA, USA). The results of cell viability, cell proliferation, and cell death assays are expressed as the mean ± standard deviation from values of independent experiments. The differences between groups were analyzed by one-way ANOVA followed by Bonferroni’s Multiple Comparison Test, as a post test. The *p* < 0.05 (*) value was considered statistically significant.

## 5. Conclusions

To the best of our knowledge, this work is the first to show in vitro evidence that a nanoemulsion containing *O. speciosa* oil or a combination with *C. langsdorffii* oil resin reduce cell viability and proliferation and modify cell morphology in primary cultures of human ectopic endometrium stromal cells, suggesting these nanoemulsions could be a new option for the endometriosis treatment.

## Data Availability

Not applicable.
